# Circumstances in a young German cohort with sudden cardiac arrest: systematic insights and implications

**DOI:** 10.1007/s00392-025-02593-9

**Published:** 2025-01-22

**Authors:** Fabienne Kreimer, Pia Thiesing, Ibrahim Akin, Jens Tiesmeier, Hendrik Milting, Andreas Mügge, Nazha Hamdani, Assem Aweimer, Ibrahim El-Battrawy

**Affiliations:** 1 University Hospital St Josef Hospital, Cardiology and Rhythmology, Ruhr University, Bochum, Germany; 2 University Hospital Münster, Rhythmology, Münster, Germany; 3 Department of Molecular and Experimental Cardiology Institut Für Forschung Und Lehre (IFL), Ruhr University Bochum, Bochum, Germany; 4 First Department of Medicine, University Medical Center Mannheim, Mannheim, Germany; 5 Institute for Anesthesiology, Intensive Care- and Emergency Medicine, MKK-Hospital Luebbecke, Campus OWL, Ruhr-University, Bochum, Germany; 6 Heart and Diabetes Center NRW, Erich and Hanna Klessmann Institute, University Hospital of the Ruhr-University Bochum, Bad Oeynhausen, Germany; 7 Department of Cardiology and Rhythmology, University Hospital St. Josef-Hospital Bochum, Ruhr University Bochum, Gudrunstraße 56, 44791 Bochum, Germany

## Abstract

**Introduction:**

Data on circumstances of sudden cardiac arrest (SCA) in Germany are limited. The present study aimed to investigate systematically the current pre- and in-hospital circumstances of a SCA cohort at young age (65 years or younger) in Germany.

**Methods:**

In the period from 2010 to 2021, we enrolled 191 consecutive patients with SCA at a university hospital in the Ruhr area, Germany. Clinical baseline characteristics and cardiopulmonary resuscitation (CPR) data were assessed.

**Results:**

A total of 191 patients (median age: 56 years (ranging from 16 to 65 years); 82% males) were included. The median duration of hospitalization was nine days. 97 patients (50.8%) deceased during hospitalization. The patients suffered SCA during non-stressful daily activities (41.4%), while working (14.7%), exercising (12.0%) or resting (8.9%). Patients experienced SCA most often at home (41.9%), in public (31.9%), at work (14.7%), or in the emergency ambulance (6.3%). Bystander-witnessed cardiac arrest was reported in 80.6% of cases. However, lay resuscitation was performed in only 46.1% of cases. The first-monitored rhythm was most frequently ventricular fibrillation (67.0%), followed by asystole (18.3%), ventricular tachycardia (5.8%), pulseless electrical activity (5.2%) and bradycardia (2.1%).

**Conclusion:**

Compared to other studies, we detected lower rates of SCA occurring at home and higher rates in public, at work or during sports. This may be related to the fact that only younger patients under the age of 65 were included in this SCA cohort.

Sirs:

Sudden cardiac arrest (SCA) is one of the most common causes of death in European countries and often affects apparently healthy people of different age groups [1]. It can occur in people with known heart disease such as coronary artery disease or structural cardiac abnormalities, but also in people without previous symptoms or known cardiac issues [1]. The SCA rate in Germany is estimated at approximately 81–122 per 100,000 inhabitants per year [2, 3]. Data on circumstances of SCA in Germany are limited. The present study aimed to investigate systematically the current pre- and in-hospital circumstances of a SCA cohort at young age (65 years or younger) in Germany. In this analysis, we focused on young patients, as they have a different risk profile than older SCA patients, and as the younger age cohort is usually underrepresented in studies and thus the available data are very limited.

In the period from 2010 to 2021, we enrolled 191 consecutive patients with SCA at a university hospital in the Ruhr area, Germany. Clinical baseline characteristics and cardiopulmonary resuscitation (CPR) data were assessed. In 40 patients the aetiology of SCA could not be recognized, e.g. for the absence of coronary angiography or further diagnostical evaluation due to death. 151 patients underwent coronary angiography and were systematically analysed regarding the cause of death. These 151 patients were then divided into groups with coronary-related (n = 111) (such as coronary artery disease, vasospasm, and coronary vascular dissection) and non-coronary-related aetiology (n = 40) (such as cardiomyopathies, cardiac channelopathies, myocarditis, and idiopathic cases). The study was approved by the local ethics committee. Data are available from the corresponding author upon reasonable request.

A total of 191 patients (median age: 56 years (ranging from 16 to 65 years); 82% males) were included. The median duration of hospitalization was nine days. 97 patients (50.8%) deceased during hospitalization. In SCA patients with non-coronary-related aetiology, the diagnosis for SCA was cardiomyopathy in 30.0% of patients (dilated: 12.5%, hypertrophic: 10.0%, others: 7.5%), myocarditis in 7.5%, valvular disease in 2.5%, long QT syndrome in 5.0% and short QT syndrome in 2.5%. In 52.5% of cases, the SCA was idiopathic.

The patients suffered SCA during non-stressful daily activities (41.4%), while working (14.7%), exercising (12.0%) or resting (8.9%). In 23.1% of cases the activity could not be recognized. Patients experienced SCA most often at home (41.9%), in public (31.9%), at work (14.7%), or in the emergency ambulance (6.3%). The location was unknown in 5.2% of cases. Bystander-witnessed cardiac arrest was reported in 80.6% of cases. However, lay resuscitation was performed in only 46.1% of cases, and within 10 min of SCA event in only 12.6%. The median time to CPR start was < 10 min. Median total duration of CPR was 31–44 min. The first-monitored rhythm was most frequently ventricular fibrillation (67.0%), followed by asystole (18.3%), ventricular tachycardia (5.8%), pulseless electrical activity (5.2%) and bradycardia (2.1%). If an external defibrillator was used, an average of three shocks were delivered. Mechanical resuscitation assistance was used in 6.3% of cases (Fig. 1, Table 1).

**Fig. 1:**
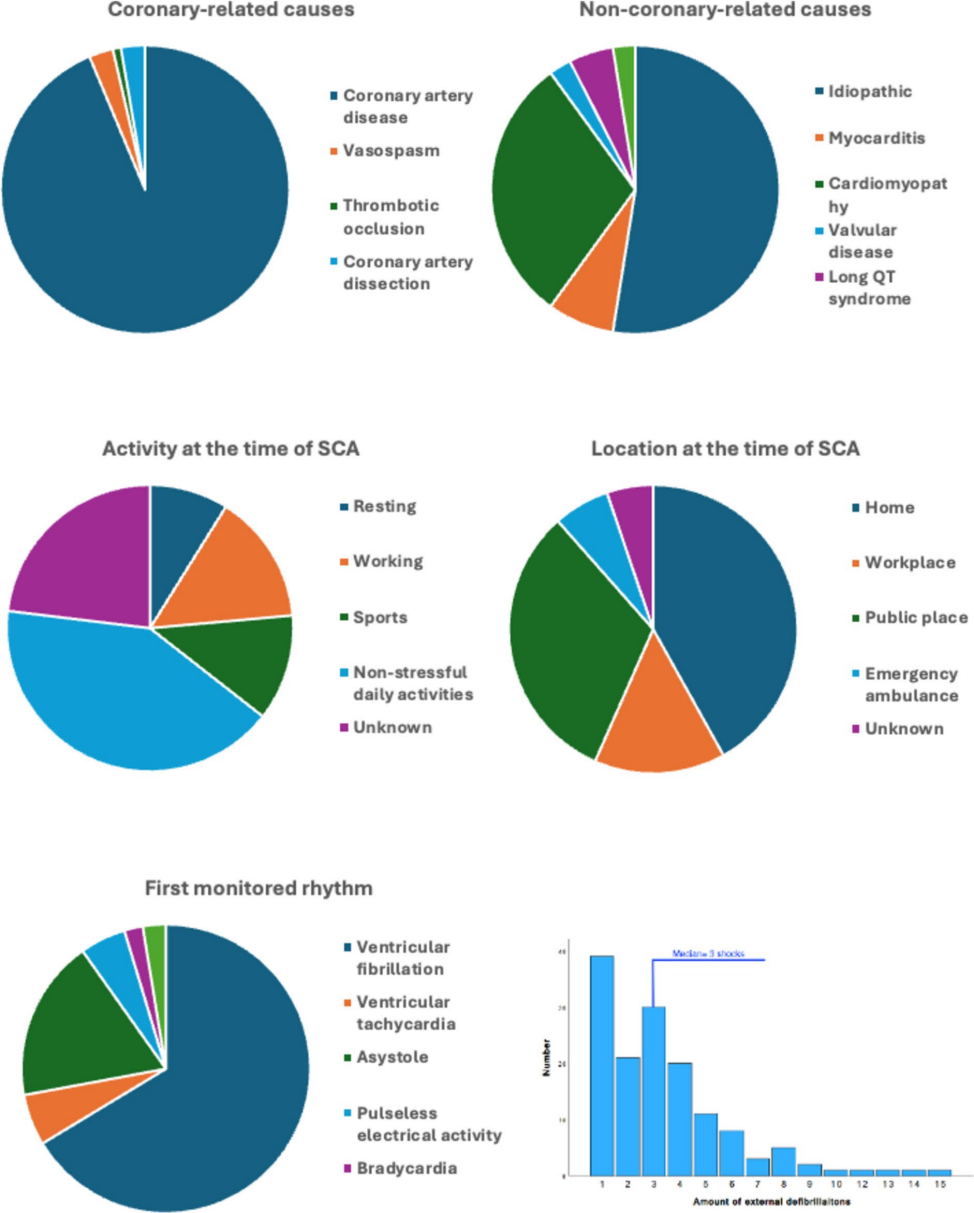
Main findings of the analysis

**Table 1:** Baseline characteristics and cardiopulmonary resuscitation data in the SCA cohort

Variables	All Patients n = 191	Coronary-related cause n = 111	Non-coronary related cause n = 40
Age –– years, median (min–max)	56 (16–65)	56 (34–65)	52 (16–65)
Male –– no. (%)	156/191 (81.7)	98/111 (88.3)	27/40 (67.5)
Duration of hospitalization –– days, median (min–max)	9 (1–144)	11 (1–109)	14 (1–144)
Deceased during hospitalization –– no. (%)	97/191 (50.8)	45/111 (40.5)	12/40 (30.0)
CPR data			
First monitored rhythm			
VF	128/191 (67.0)	83/111 (74.8)	29/40 (72.5)
VT	11/191 (5.8)	7/111 (6.3)	4/40 (10.0)
Asystole	35/191 (18.3)	15/111 (13.5)	6/40 (15.0)
Pulseless electrical activity	10/191 (5.2)	2/111 (1.8)	2/40 (5.0)
Bradycardia (< 40 bpm)	4/191 (2.1)	2/111 (1.8)	1/40 (2.5)
Unknown	5/191 (2.6)	2/111 (1.8)	0/40 (0)
Use of defibrillator	146/191 (76.4)	98/111 (88.3)	32/40 (80.0)
If yes: Number of shocks delivered –– median (min–max)	3 (1–15)	3 (1–15)	1.5 (1–12)
CPR in which situation/mechanism			
Resting	17/191 (8.9)	8/111 (7.2)	5/40 (12.5)
Sports	23/191 (12.0)	12/111 (10.8)	6/40 (15.0)
Non-stressful daily activities	79/191 (41.4)	46/111 (41.4)	15/40 (37.5)
Working	28/191 (14.7)	20/111 (18.0)	4/40 (10.0)
Unknown	44/191 (23.1)	25/111 (22.5)	10/40 (25.0)
Use of mechanical resuscitation assistance	12/191 (6.3)	6/111 (5.4)	0/40 (0)
Location at cardiac arrest – no. (%)			
Home	80/191 (41.9)	45/111 (40.5)	18/40 (45)
Workplace	28/191 (14.7)	19/111 (17.1)	6/40 (15.9)
P ublic place	61/191 (31.9)	34/111 (30.6)	14/40 (35.0)
Emergency ambulance	12/191 (6.3)	5/111 (4.5)	1/40 (2.5)
Hospital	–	–	–
Unknown	10/191 (5.2)	8/111 (7.2)	1/40 (2.5)
Bystander-witnessed cardiac arrest	154/191 (80.6)	92/111 (82.9)	34/40 (85.0)
Layman- performed CPR	88/191 (46.1)	52/111 (45.8)	22/40 (55.0)
Time to start CPR (in minutes)			
Median (min–max)	< 10 (< 10 to ‘21–30’)	< 10 (< 10 to 21–30)	< 10 (< 10 to 11–20)
Total duration of CPR (min.)			
Median (min–max)	31–44 (< 10 to > 60)	31–44 (< 10 to > 60)	21–30 (< 10 to > 60)
Pre-hospital duration CPR (min.)			
Median (min–max)	21–30 (< 10 to > 60)	21–30 (< 10 to > 60)	11–20 (< 10 to > 60)
≤ 10 min	24/191 (12.6)	12/111 (10.8)	9/40 (22.5)
> 10 min	133/191 (69.6)	77/111 (69.4)	26/40 (65.0)
Unknown pre-hospital CPR duration (%)	34/191 (17.8)	22/111 (19.8)	5/40 (12.5)
Intra- hospital duration CPR (min.)			
Median (min–max)	21–30 (< 10 to > 60)	21–30 (< 10 to > 60)	11–20 (< 10 to > 60)
≤ 10 min	11/191 (5.8)	4/111 (3.6)	4/40 (10.0)
> 10 min	62/191 (32.5	28/111 (25.2)	5/40 (12.5)
Unknown intra-hospital CPR duration (%)	123/232 (53.0)	79/111 (71.2)	31/40 (77.5)
Neurological outcome per GOS:			
1 = Death	97/191 (50.8)	45/111 (40.5)	12/40 (30)
2 = Persistent Vegetative State	2/191 (1.0)	2/111 (1.8)	0/40 (0)
3 = Severe Disability	5/191 (2.6)	3/111 (2.7)	2/40 (5.0)
4 = Moderate Disability	8/191 (4.2)	7/111 (6.3)	1/40 (2.5)
5 = Good recovery	77/191 (40.3)	53/111 (47.7)	24/40 (60.0)
Unknown	2/191 (1.0)	1/111 (0.9)	1/40 (2.5)

*Bpm* beats per minute; *CPR* cardiopulmonary resuscitation; *GOS* Glasgow Outcome Scale; *VF* ventricular fibrillation; *VT* ventricular tachycardia

In patients with coronary-associated SCA, ventricular fibrillation was more common, and ventricular tachycardia, asystole, and pulseless electrical activity were less common compared to patients with non-coronary-associated causes, however, without statistical significance. Additionally, the average shock delivery was twice as high (3 shocks vs. 1.5 shocks). Interestingly, layperson-initiated CPR was more frequent in patients with non-coronary-associated causes (55.0% vs. 45.8%), despite comparable rates of bystander-witnessed cardiac arrest (85.0% vs. 82.9%). It is also noteworthy that the mean CPR duration, overall as well as prehospital and in-hospital, was shorter in patients with non-coronary-associated causes (21–30 min vs. 31–44 min). Differences were also observed in the outcome. More patients with coronary causes died than patients with non-coronary causes (40.5% vs. 30.0%) and patients with coronary causes were less likely to have a good recovery (47.7% vs. 60.0%).

Compared to other studies, we detected lower rates of SCA occurring at home and higher rates in public, at work or during sports [4, 5]. This may be related to the fact that only younger patients under the age of 65 were included in this SCA cohort.

Within the last decade, layperson resuscitation in Germany has significantly increased; however, it remains below the European average [5]. Additionally, it is concerning that the lay resuscitation rate in the Ruhr area is notably lower than the rate reported by the German Resuscitation Registry (46.1% vs. 51.3%) [5].

Fewer patients with non-coronary causes died, which could be due to a higher rate of layperson CPR, a shorter delay in starting CPR and a shorter overall duration of CPR. This may partly explain why the neurological outcome of patients with non-coronary causes was significantly better.

The study has limitations. Firstly, it is based on single-center observations. In addition, the cohort size was relatively small, although it represents the largest systematic investigation of the circumstances of an SCA cohort in Germany to date. Another limitation is the lack of information on the total number of SCAs that occurred in the region during the study period but were either not admitted to the hospital or admitted to another hospital. This results in a potential selection bias as the analysis is limited to cases that were successfully resuscitated and transferred to our hospital.

In conclusion, this study of a German young SCA cohort demonstrates that cardiac arrest occurred most frequently at home or in public during non-stressful daily activities and was witnessed in most cases. The lay resuscitation rate was significantly below the European and national average. These rates have to be increased in the future by raising awareness of the condition, promoting CPR, and improving access to automated external defibrillators in public places.

## Abbreviations

CPR: Cardiopulmonary resuscitation
SCA: Sudden cardiac arrest
