# Publisher Correction: Circumstances in a young German cohort with sudden cardiac arrest: systematic insights and implications

**DOI:** 10.1007/s00392-025-02621-8

**Published:** 2025-03-06

**Authors:** Fabienne Kreimer, Pia Thiesing, Ibrahim Akin, Jens Tiesmeier, Hendrik Milting, Andreas Mügge, Nazha Hamdani, Assem Aweimer, Ibrahim El-Battrawy

**Affiliations:** 1 University Hospital St Josef Hospital, Cardiology and Rhythmology, Ruhr University, Bochum, Germany; 2 University Hospital Münster, Rhythmology, Münster, Germany; 3 Department of Molecular and Experimental Cardiology Institut Für Forschung Und Lehre (IFL), Ruhr University Bochum, Bochum, Germany; 4 First Department of Medicine, University Medical Center Mannheim, Mannheim, Germany; 5 Institute for Anesthesiology, Intensive Care- and Emergency Medicine, MKK-Hospital Luebbecke, Campus OWL, Ruhr-University, Bochum, Germany; 6 Heart and Diabetes Center NRW, Erich and Hanna Klessmann Institute, University Hospital of the Ruhr-University Bochum, Bad Oeynhausen, Germany; 7 Department of Cardiology and Rhythmology, University Hospital St. Josef-Hospital Bochum, Ruhr University Bochum, Gudrunstraße 56, 44791 Bochum, Germany

**Publisher Correction: Clinical Research in Cardiology** 10.1007/s00392-025-02593-9

The article "Circumstances in a young German cohort with sudden cardiac arrest: systematic insights and implications", written by Fabienne Kreimer, Pia Thiesing, Ibrahim Akin, Jens Tiesmeier, Hendrik Milting, Andreas Mügge, Nazha Hamdani, Assem Aweimer, Ibrahim El Battrawy, was originally published under exclusive license to Springer-Verlag GmbH Germany, part of Springer Nature, because of a malfunction in our production system The article should have been published under the open access model.

Therefore the article’s copyright notice was changed on 5 February 2025 to © The Author(s) 2025 and the article is now distributed under a Creative Commons Attribution 4.0 International License, which permits use, sharing, adaptation, distribution and reproduction in any medium or format, as long as you give appropriate credit to the original author(s) and the source, provide a link to the Creative Commons licence, and indicate if changes were made.

The images or other third party material in this article are included in the article’s Creative Commons licence, unless indicated otherwise in a credit line to the material. If material is not included in the article’s Creative Commons licence and your intended use is not permitted by statutory regulation or exceeds the permitted use, you will need to obtain permission directly from the copyright holder. To view a copy of this licence, visit http://creativecommons.org/licenses/by/4.0.

Open Access funding enabled and organized by Projekt DEAL.

The Publisher apologises for the mistake.

